# Screening of biobank SNP-array genotyping data to detect Lynch syndrome predisposing *MLH1* copy number variants

**DOI:** 10.1007/s10689-025-00476-6

**Published:** 2025-05-26

**Authors:** Kimmo Ala-Kulju, Olli Carpén, Maarit Lappalainen, Minja Pehrsson

**Affiliations:** 1 Helsinki Biobank, Helsinki University Hospital, Helsinki, Finland; 2 Department of Pathology, HUS Diagnostic Center, Helsinki University Hospital, Helsinki, Finland; 3 Department of Pathology, Medicum, University of Helsinki, Helsinki, Finland; 4 Laboratory of Genetics, HUS Diagnostic Center, Helsinki University Hospital, Helsinki, Finland

**Keywords:** Biobank, Genetic screening, Genotyping data, Lynch syndrome, *MLH1*, Copy number variant, Personalized medicine

## Abstract

Efficient use of genetic biobank data in support of clinical care would enhance the adoption of personalized medicine. Identification of carriers of medically actionable variants that predispose to cancer enables intensified screening and follow-up to decrease disease risk. Pathogenic variants of the *MLH1* gene cause Lynch syndrome with a significant risk of developing cancer. Here, we introduce a novel approach for the large-scale screening of biobank SNP-array-based genotyping data to analyze copy-number variants (CNVs). With the method developed, we analyzed the Helsinki Biobank cohort of 121 073 samples and identified 29 *MLH1* exon 16 deletion (*MLH1*∆Ex16) carriers, of which five (17%) had not been previously identified in healthcare. Our results demonstrate a high positive predictive value for the identification of *MLH1*∆Ex16 carriers from genotyping data. The cost-efficient method for detection of CNV carriers from large biobank genotyping cohorts described here facilitates intensified screening and follow-up aiming to cancer prevention.

## Introduction

Biobanks collect, process, store and distribute biological samples and associated health data to support medical research. Biobanks have become a significant source of genetic information by accumulating genomic data generated in research studies. Biobanking can significantly contribute to personalized medicine and support patient care if medically actionable findings, gained from biobank data, are utilized efficiently in healthcare. Opportunistic screening of biobank sample data could dramatically enhance the implementation of personalized and preventive medicine.

Lynch syndrome (LS), also known as hereditary nonpolyposis colorectal cancer (HNPCC), predisposes to early onset-cancer, mainly of colorectal, endometrial and ovarian origin, but also to other cancers. The autosomal dominant syndrome is caused by germline mutations in DNA mismatch repair genes *MLH1*, *MSH2*, *MSH6* and *PMS2* or deletions in the non-mismatch repair gene *EPCAM*. The incidence of LS predisposing pathogenic variants has been estimated to approximately 1:300 [1]. In Finland, most LS cases are caused by pathogenic variants in *MLH1*, of which the most common is the *MLH1* exon 16 deletion (*MLH1*∆Ex16) founder variant where the entire exon 16 has been lost as a result of Alu-mediated recombination [2]. Screening for pathogenic *MLH1* variants is clinically important, as the cumulative risk of developing colorectal cancer before the age of 70 is 53% for male carriers and 42% for female carriers, while the risk for developing endometrial cancer is 36% and the risk of ovarian cancer is 8% [3]. Identification of healthy variant carriers would enable intensified screening and follow-up aimed at early detection of tumors and reduced cancer mortality [4]. Lynch syndrome predisposing genes have been categorized by the Centers for Disease Control and Prevention screening group to Tier 1 as the most appropriate genes for screening, with high penetrance, well-understood links to disease and effective interventions for prevention or mitigation of disease risk [5].

Genotyping with single nucleotide polymorphism (SNP) -arrays has been the most commonly used method for producing genetic information from biobank samples due to its relatively low cost. The large FinnGen biobank research project has generated such genotyping data from 520 000 biobank donors [6]. However, array-based genotyping data is not optimal for the reliable detection of very rare variants [7] or copy number variants (CNVs) of single exons [8]. To enable CNV analysis from SNP-array data, methods utilizing SNP-array intensity values have been developed [9]. The objective of this study was to build an efficient and scalable approach to detect rare CNVs suitable for large-scale screening of biobank SNP-array data. By developing a method for detecting the *MLH1*∆Ex16 variant from the Helsinki Biobank (HBB) FinnGen cohort, we strived to identify individuals predisposed to an elevated risk of cancer for whom preventive measures could be provided.

## Materials and methods

### Study data

The data for this study consists of SNP-array genotyping data from 121 073 Helsinki Biobank samples, produced in the FinnGen project (www.finngen.fi/en). Genotyping of blood-derived DNA was performed using FinnGen ThermoFisher Axiom custom array v1 (*N* = 34 504; 28% of the samples) and v2 (*N* = 86 569; 72% of the samples) as described elsewhere [6]. The unique probe set content in v1 and v2 arrays are 760 904 and 723 375, respectively, with an overlap of 708 552 probe sets between the arrays based on chip manifests. The protocol for this study was approved by the Helsinki University Hospital (HUS) Regional Committee on Medical Research Ethics (HUS/428/2024).

### Array data Preparation

*MLH1* exon 16 deletion, NM_000249.4 (MLH1):c.1731+2247_1897–402del (ClinVar variation ID 1332889), is a 3 538 base pair (bp) in-frame deletion at chromosomal location 3:37044575–37048112 (GRCh38). Both FinnGen genotyping arrays (v1 and v2) contain a total of 21 probe sets for 18 unique loci in the deletion region. In addition to these, probe sets for 50 loci flanking both sides of the deletion region were included in the analysis. To allow combining the data from both array types, only overlapping probe sets present on both arrays were considered in the flanking regions.

The intensity values for both alleles of the selected probe sets were extracted from the raw array data CEL files with Analysis Power Tools (APT) Release 2.12.0 (Thermo Fisher Scientific). The appropriate tools from the software package were run without variant calling steps to only extract intensity values for all the samples. The software performed default artefact removal, probe summarization and normalization steps for the extracted intensities.

### Analysis of MLH1 exon 16 deletion

For each sample, the sum of the intensities of both alleles of all probe sets interrogating the same locus was calculated to represent the total chromosomal signal intensity at that position. These locus-wise summed values were further quantile normalized with respect to the standard normal distribution over all samples.

To identify the samples with *MLH1*∆Ex16, two features were used for cluster analysis: the difference between median intensity of the deletion and flanking regions, and the median absolute deviation (MAD) of the intensity values. MAD was calculated in a piecewise manner over the deletion and flanking regions. As a robust measure of dispersion, MAD feature was included to aid in differentiating the noisy unclassifiable samples characterized by large within-sample intensity variances.

Given the unbalanced cluster sizes due to the rarity of the deletion, typical clustering algorithms tended to work inconsistently and require a lot of parameter fine-tuning. Preferably, additional datasets would be needed to validate the choice of the algorithm and its parameters. In the absence of multiple datasets, simple thresholding rules based on the visual inspection of the cluster plot were used to determine samples with suspected deletions.

### Confirmation of variant carriers, electronic health record (EHR) review and sensitivity assessment

For confirmation of the results, a manual detailed EHR review was conducted of the identified putative *MLH1*∆Ex16 carriers to assess whether the deletion had been previously identified by diagnostic testing in health care. The variant status for sample donors suspected with the deletion but without existing information of the deletion variant, *MLH1*, LS or HNPCC in EHR were validated from existing HBB DNA samples with a polymerase chain reaction (PCR) assay in the accredited genetic diagnostic laboratory of HUS Diagnostic Center. The demographics of identified *MLH1*∆Ex16 carriers were collected from EHR, and personal history of cancer was determined using EHR-extracted International Classification of Diseases (ICD)-10 codes.

The sensitivity of the method was assessed by conducting a search for diagnostically determined *MLH1*∆Ex16 carriers in the hospital EHR database, covering all individuals from whom genotyping information was available. The search covered unstructured free-text medical notes and statements. Text snippets with short context that included the *MLH1* gene name with common spelling variants were extracted from the EHR databases and manually reviewed to identify diagnosed *MLH1*∆Ex16 carriers and individuals confirmed negative for the deletion.

## Results

### Identification and validation of *MLH1* exon 16 deletion samples

**Fig. 1:**
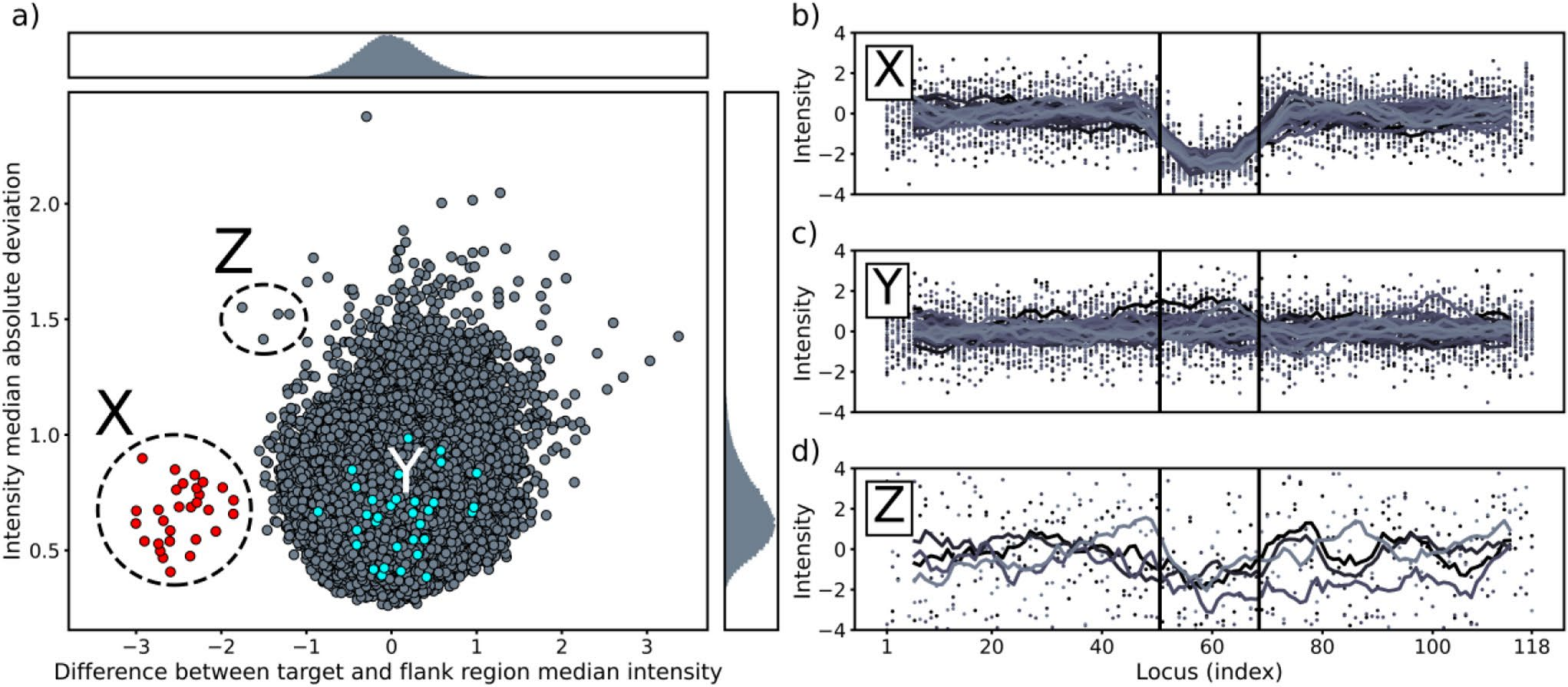
Results of the intensity analyses of *MLH1* exon 16. (**a**) Two-feature cluster plot of all analyzed samples with marginal histograms on the sides. Samples suspected to contain heterozygous *MLH1* exon 16 deletion (red) form a separate cluster (X) on the left side of the main cluster. Samples diagnostically determined as negative for the deletion are indicated in cyan color (Y). Examples of outlier samples (Z) to the main cluster are indicated. (**b**) All the identified 29 samples with suspected *MLH1* exon 16 deletion from the cluster X. Variant total signal intensities in consecutive loci of the examined regions are plotted. Singular intensity values are depicted as dots and the squiggly lines are their per-sample rolling averages with a window size of 10. The target deletion region is between the two black vertical lines. (**c**) 32 samples (Y) diagnostically determined as negative for the deletion from the main cluster. Location of these samples is indicated with color cyan in the cluster plot. (**d**) Four arbitrarily selected examples (Z) of ambiguous noisy samples with a high median absolute deviation in the intensity values

Out of 121 073 genotyped HBB sample donors, 29 (0.024%) individuals were identified as putative heterozygous *MLH1*∆Ex16 carriers based on the cluster plot of intensity features (Fig. 1a). The suspected deletion samples (X) with abnormal difference between the deletion and flanking region median intensities, but low overall intensity MAD, formed a visually distinguishable low-density cluster of outliers on the left side of the main cluster. For these samples, the locus-wise variant intensity plot showed the characteristic drop in total intensities within the deleted region compared to the flanking regions (Fig. 1b). In comparison, samples confirmed negative for the deletion by diagnostic tests (Y) are located in the main cluster and did not show a similar drop in the intensities (Fig. 1c). Signal intensities for some of the outliers (Z) to the main cluster are also demonstrated (Fig. 1d) as examples of noisy and ambiguous high-variance samples.

**Fig. 2:**
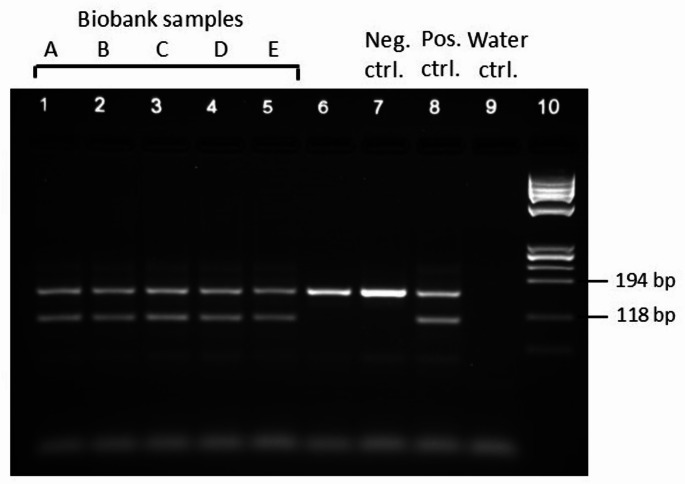
Validation of *MLH1* exon 16 deletion of the suspected deletion samples without EHR confirmation. PCR-assay of the five biobank samples (A–E) with suspected *MLH1* exon 16 deletion. A deletion specific amplification product of 113 bp identifies the mutant allele in all five biobank samples in addition to the wild-type allele of 167 bp. Line 6 represents a diagnostic patient sample without the *MLH1* exon 16 deletion. Neg. ctrl: negative control, pos. ctrl: positive control

To evaluate the reliability of the method, all potential deletion samples (Fig. 1a cluster X and Fig. 1b) were further investigated to confirm the mutation. Manual EHR review was conducted to assess previous diagnostic testing performed in health care. Of the identified 29 individuals, 24 (83%) had a record of the *MLH1* exon 16 deletion. The five samples, for which there were no mention in EHR, were further analyzed with a diagnostic PCR-assay for the deletion. All five samples were confirmed as true *MLH1*∆Ex16 carriers (Fig. 2) demonstrating a 100% positive predictive value (PPV) for the method.

To estimate the sensitivity of the method, an EHR database search for the whole analysis cohort was conducted. The aim was to discern *MLH1*∆Ex16 carriers known to the healthcare system but not identified in the analysis (false negatives). No such cases were found indicating a high sensitivity for the method. However, the text search using *MLH1* as search term flagged only 16/24 (67%) of the confirmed *MLH1*∆Ex16 carriers identified in the analysis.

### Clinical characteristics of identified *MLH1* exon 16 deletion carriers

The mean age of the identified *MLH1*∆Ex16 carriers was 62 years with a range of 32–85 years, including 14 females and 15 males. Based on EHR review, 22/29 (76%) had been assigned at least one ICD-10 group C (cancer) diagnosis and 11/29 (38%) had more than one cancer diagnosis. As expected, the most common cancer diagnosis among *MLH1*∆Ex16 carriers was malignant neoplasms of the colon in 38% of cases (Table 1). Of the five *MLH1*∆Ex16 carriers that had not been identified in healthcare, three had no cancer diagnosis recorded in EHR.

**Table 1:** Primary cancer diagnoses of the identified individuals with *MLH1* exon 16 deletion

ICD-10 code	Description	Count
**C08**	Malignant neoplasm of other and unspecified major salivary glands	1
**C17**	Malignant neoplasm of small intestine	3
**C18**	Malignant neoplasm of colon	11
**C20**	Malignant neoplasm of rectum	2
**C25**	Malignant neoplasm of pancreas	3
**C37**	Malignant neoplasm of thymus	1
**C44**	Other malignant neoplasms of skin	5
**C48**	Malignant neoplasm of retroperitoneum and peritoneum	1
**C50**	Malignant neoplasm of breast	3
**C53**	Malignant neoplasm of cervix uteri	1
**C54**	Malignant neoplasm of corpus uteri	1
**C56**	Malignant neoplasm of ovary	1
**C61**	Malignant neoplasm of prostate	3
**C64**	Malignant neoplasm of kidney, except renal pelvis	1
**C67**	Malignant neoplasm of bladder	2
**C91**	Lymphoid leukaemia	1

Numbers are based on ICD-10 group C diagnoses assigned to identified *MLH1*∆Ex16 carriers (*N* = 29). 11 individuals had more than one cancer diagnosis assigned to them and are counted in multiple rows

## Discussion

In this study, we developed a scalable method that enables screening of rare CNVs from SNP-array genotyping data. Using this approach, we aimed at identifying carriers of the most common Finnish LS predisposing *MLH1* variant, deletion of exon 16, from the cohort of over 120 000 HBB sample donors. Our results demonstrate a high PPV (100%) for detecting *MLH1*∆Ex16 carriers from SNP-array genotyping data, which is a crucial property for avoiding the high costs associated with validation of false positive samples in biobank-scale. A previous study conducted on the FinnGen data utilizing haplotype proxy to identify *MLH1*∆Ex16 carriers achieved a PPV of 22% [10]. Based on our results, the accuracy can be significantly improved by incorporating intensity data to the analyses.

The 0.024% prevalence of *MLH1*∆Ex16 carriers in the HBB cohort is in line with previous estimates of 0.051% [1] which included also other pathogenic *MLH1* variants. The majority, 83% of the variant carriers, had been previously identified in healthcare, indicative of efficient cascade testing in Finnish Lynch families facilitated by the nationwide LS registry. Nonetheless, five individuals without previous knowledge of their carrier status were identified from the biobank cohort, demonstrating that opportunistic screening of biobank samples enables identification of also LS carriers undiagnosed in healthcare. Prevalence of cancer diagnoses among the carriers was high (76%), reflecting the clinical significance of the variant, but also the fact that hospital patients are concentrated in HBB sample donors.

The subjective cluster determination can be regarded as a limitation of the study. Development of a more objective algorithmic cluster analysis will be feasible in the future as the number of datasets increases and other CNVs are investigated. Parallel validation of likely negative but near-borderline and high-variance samples will also bring more reliability to the method. Although the analysis indicated a high sensitivity for the approach as no false negative samples were recognized by EHR searches, challenges to confidently assess the sensitivity remain. EHR search on unstructured data for large cohorts has its own inherent inaccuracies, and not all true identified *MLH1*∆Ex16 carriers were caught up using only *MLH1* as the search term. In light of this, true deletion carriers might have gone unrecognized in the EHR search. Obviously, also *MLH1*∆Ex16 carriers unknown in healthcare are not covered in the sensitivity assessment. Monitoring of the sensitivity of the method will continue.

Genetic biobank data can be used in personalized disease risk screening and preventive medicine to identify individuals at risk of disease. Our results demonstrate that there is a clinical benefit of screening for LS predisposing gene variants from biobank samples, as carriers previously undiagnosed could be identified, even before the development of cancer. Results will be offered to the identified *MLH1*∆Ex16 carriers as part of the RETURN-study. The approach described here can be applied not only for LS carriers, but also expanded to the detection of other CNVs of medical significance. By identifying clinically actionable variant carriers and returning the research results to biobank donors, biobanking has the potential to greatly enhance personalized medicine in a cost-efficient way and enable disease prevention.

## Data Availability

No datasets were generated or analysed during the current study.
